# Observations on Relaxed Rectum

**Published:** 1845-12

**Authors:** Henry Hunt


*Observations on Relaxed Rectum.* By Henry Hunt, m. d.—The author of the paper on this subject observes, that the most prominent symptoms of the affection are obstinate constipitation, with tenesmus, the sensation of a load in the lower bowel, which is not relieved by an evacuation, and the discharge of a bloody mucus. The irritation frequently extends to the genito-urinary organs. On examination the rectum is found to be preternaturally enlarged, and more or less filled with large folds of mucous membrane, which impede both the passage of the fæces and the introduction of the instruments. The consequences of mismanagement or neglect of this condition of the bowel are stated to be prolapsus ani, with irritable sphincter, and a species of intussusception of the upper and undilated part of the bowel into the lower and dilated part. The causes of the affection are habitual inattention to the calls of nature, and consequent over-distension of the gut. The treatment recommended by the author, and also by Mr. Bransby Cooper, consists in the avoidance of all apperient medicines, and the injection of a pint of cold water into the bowel every night previous to going to bed; the removal of the prolapsus, and the application of belladonna ointment to the irritable sphincter. A practical injunction is given by Mr. Cooper, to the effect that in all diseases of the rectum, the bowels should be relieved if possible at night instead of morning; because that in the latter case, the moving about during the day tended to displace the bowel, whilst that accident is prevented by repose in bed, after passing an evacuation.—*Paper read before the Medico-Chirurgical Society.—Ibid.*

